# A Gait-Conditioned Human-in-the-Loop Optimization Framework Based on Gait–Assistance–Response Relationship Model for Exoskeleton Assistance

**DOI:** 10.3390/bioengineering13101210

**Published:** 2026-10-16

**Authors:** Jiaqi Han, Liping Wang, Jianquan Ding, Yuzhen Zhang, Xuelun Liu, Qichong Bian, Zhibo Jing, Juanjuan Zhang

**Affiliations:** 1 College of Artificial Intelligence, Nankai University, Tianjin 300350, China; hanjiaqi@mail.nankai.edu.cn (J.H.); lipingwang@mail.nankai.edu.cn (L.W.); jianquanding@mail.nankai.edu.cn (J.D.); zhangyuzhen@mail.nankai.edu.cn (Y.Z.); liuxuelun@mail.nankai.edu.cn (X.L.); bianqc@mail.nankai.edu.cn (Q.B.); 2 Tianjin Key Laboratory of Intelligent Robotics, Institute of Robotics and Automatic Information System, Nankai University, Tianjin 300350, China; 3 Academy for Advanced Interdisciplinary Studies, Nankai University, Tianjin 300350, China

**Keywords:** knee exoskeleton, gait condition, assistance pattern, human-in-the-loop optimization, hierarchical relationship model

## Abstract

The effectiveness of wearable exoskeletons critically depends on assistance patterns, yet their determination is challenging because physiological responses vary across individuals and gait conditions. Human-in-the-loop (HIL) optimization can personalize assistance, but its high experimental cost limits its application across diverse tasks. In this study, we developed a gait-conditioned HIL optimization framework based on a hierarchical gait–assistance–response model. The model characterizes assistance–response relationships using parameterized surfaces and predicts gait-dependent surface parameters through polynomial regression. The experiments focused on knee exoskeletons with extension assistance, where the physiological response was quantified as muscle activation based on normalized sEMG RMS from three major knee extensors on the assisted side. The model was built on data collected under predefined gait conditions and used to guide HIL optimization in validation experiments involving five healthy adults under unseen gait conditions. The validation results showed that, with the same CMA-ES optimizer but different configurations, two-generation model-guided HIL optimization showed a mean additional 6.4 percentage-point reduction in muscle activation compared with two-generation empirical HIL optimization, approaching four-generation empirical HIL optimization with only a mean 1.3 percentage-point difference. The preliminary results suggest the potential of the proposed strategy to reduce the number of optimization generations while maintaining comparable assistance performance by providing guidance for optimization configurations.

## 1. Introduction

Wearable robotic exoskeletons have demonstrated significant potential in enhancing human locomotor capabilities [1,2,3,4] and facilitating gait rehabilitation [5,6,7]. For example, knee exoskeletons can partially substitute muscle function by providing external torque assistance during walking, thereby reducing physical effort and improving locomotor efficiency [4,8,9,10]. In the field of gait assistance, reductions in relevant muscle activation have been widely adopted as an important metric for performance evaluation [11,12,13].

Assistance patterns significantly influence physiological responses to exoskeleton assistance [14], with appropriate timing and magnitude being critical factors in achieving effective assistance benefits [15,16]. However, the physiological effects induced by a given assistance pattern are not invariant but vary across users [17,18] and gait conditions [19,20,21]. This variability highlights the necessity of customizing assistance patterns to the current user and gait condition.

To achieve assistance pattern customization, one common approach is to incorporate human feedback into exoskeleton control. Previous studies have generated desired assistance profiles based on real-time physiological measurements and biomechanical estimation [22,23,24], enabling adaptive assistance. However, the mapping between human feedback and assistance profiles is typically predefined, limiting the capability of personalization. Human-in-the-loop (HIL) optimization is a widely used approach for achieving personalized exoskeleton assistance patterns [1,15,25,26,27]. This approach measures the user’s real-time physiological responses under periodically changing assistance patterns and updates the assistance parameters iteratively to optimize a physiological response function.

However, the long optimization time limits the broader application of HIL optimization. Metabolic cost-based approaches [1,2,28] often require more than one hour, while faster objectives based on muscle activation [13,15,29] still require tens of minutes to complete. Moreover, the optimized patterns are gait-dependent. Even for the same user, the optimal parameters can shift substantially when gait conditions change [19,30]. These limitations restrict the feasibility of individually customizing assistance patterns across different gait conditions.

To reduce the burden of customizing gait-dependent assistance patterns, several studies have explored data-driven approaches based on prior experimental data. Some studies [21,31] estimated optimal assistance parameters under different gait conditions through systematic scanning experiments; however, the tested gait conditions were limited, and generalization to unseen conditions was not investigated. A prior study [2] developed a data-driven model to enable gait-dependent optimization and improved time efficiency, although establishing such a model required extensive experimental data. Another lightweight model [30] captured the relationship between gait conditions and optimized assistance patterns to provide gait-dependent initial guesses for optimization. However, further improvements may be achieved by exploiting the additional information contained in the collected data.

The aim of this study is to develop a gait-conditioned framework to guide the configuration of HIL optimization under unseen gait conditions. The framework is designed to achieve two related goals: (1) reducing the optimization burden without substantially compromising assistance effectiveness; (2) and improving assistance performance when only a limited online evaluation budget is available. The framework is based on a hierarchical model, which characterizes the relationship among gait conditions, assistance patterns, and physiological responses. Focusing on knee exoskeleton extension assistance, we conducted 6 × 6 parameter scanning experiments under 12 predefined gait conditions for data collection. Based on the dataset, a parameterized response surface model was selected to represent assistance–response relationship under individual gait conditions. Then, a regression model was used to establish the relationship between gait conditions and surface parameters. Afterwards, the proposed model predicted response landscapes under validation conditions, providing guidance for configuring HIL optimization. Experimental validation with multiple participants demonstrated that the proposed framework achieved comparable assistance benefits with substantially fewer optimization iterations under the test conditions.

## 2. Model-Guided HIL Optimization Framework

**Figure 1:**
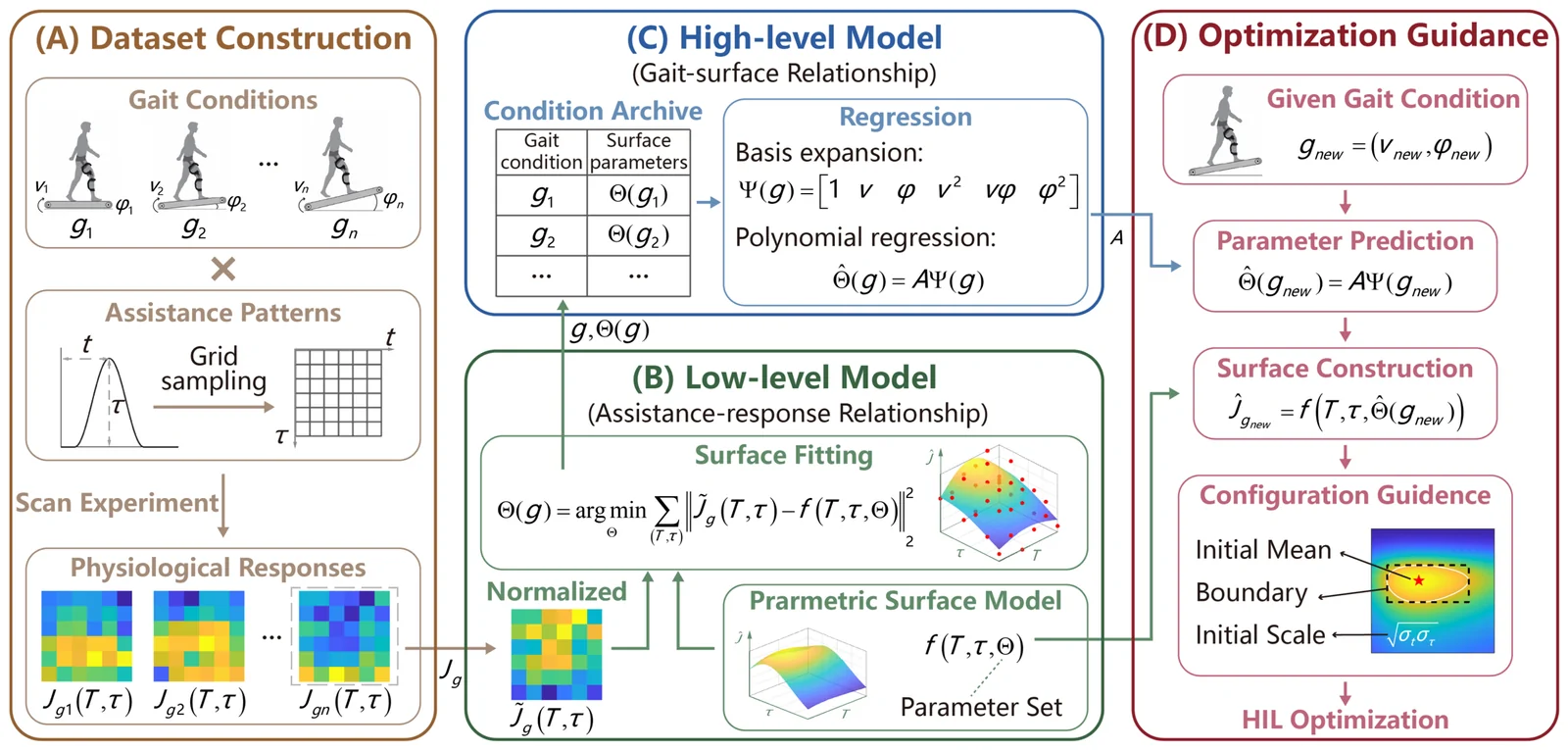
Overview of the model-guided HIL optimization Framework. (**A**) Physiological response scan experiments under multiple gait conditions and assistance patterns provide the dataset for model construction. (**B**) The low-level model formulates the relationship between assistance patterns and physiological responses under each gait condition using a parameterized surface model. (**C**) The high-level model aggregates data across multiple gait conditions, and establishes the mapping from gait conditions to surface parameters via global regression. (**D**) The learned model is used to guide optimization configurations under given gait conditions.

The proposed framework consists of data collection, hierarchical modeling, and optimization guidance, as illustrated in Figure 1. Physiological response data are collected through multi-gait scan experiments. The hierarchical model includes a low-level model that represents the assistance–response relationship under each gait condition using parameterized surfaces, and a high-level model that maps gait conditions to surface parameters. The proposed framework predicts gait-conditioned assistance landscapes and guides the configuration of optimization algorithms.

### 2.1. Dataset Construction

The construction of the hierarchical model relies on physiological response data collected through multi-gait scan experiments, where the assistance parameter space is systematically explored (Figure 1A).

The gait condition is defined by walking speed *v* and slope ϕ, denoted as g=(v,ϕ). The assistance pattern is parameterized by assistance timing *T* and assistance magnitude τ. For each selected gait condition *g*, a scan experiment is conducted to systematically evaluate physiological responses over the assistance parameter space. The scan range was defined as $T\in [T_{min},T_{max}]$ and $\tau \in [\tau_{min},\tau_{max}]$. The resulting physiological response function value is denoted as $J_{g}\left(T,\tau \right)$.

To mitigate scale variation derived from experimental conditions, the response function is normalized within each scan experiment using min–max scaling, yielding the normalized physiological response function ${\tilde{J}}_{g}\left(T,\tau \right)$. A standard or reversed normalization scheme is applied depending on whether higher or lower values indicate better assistance effectiveness.

### 2.2. Assistance–Response Relationship Model

Based on the physiological response data collected from scan experiments, a parametric modeling framework is proposed to characterize the relationship between assistance parameters and physiological responses under different gait conditions, as illustrated in Figure 1B.

We adopt a Gaussian-inspired parametric formulation as a structural prior for modeling the assistance–response relationship, and accordingly define a family of smooth and convex response surfaces. These surfaces are parameterized, and each model instance is characterized by a distinct parameter set Θ. The detailed formulations are given as follows:

#### 2.2.1. Standard Gaussian (G)

The baseline model adopts a standard two-dimensional Gaussian formulation with independent timing and magnitude dimensions.

(1) $$\Theta ={\left[\begin{array}{cccc} \;T^{*} & \tau^{*} & \sigma_{T} & \sigma_{\tau }\; \end{array}\right]}^{T}$$

where $T^{*}$ and $\tau^{*}$ represent the centering locations of assistance timing and magnitude, respectively, and $\sigma_{T}$ and $\sigma_{\tau }$ are the corresponding scale parameters in the normalized parameter-deviation space. To avoid mixing the physical units of assistance timing and assistance magnitude, the parameter deviations were normalized as:

(2) $$\Delta T=\frac{T-T^{*}}{T_{max}-T_{min}},\;\Delta \tau =\frac{\tau -\tau^{*}}{\tau_{max}-\tau_{min}}$$

The modeled physiological response function is then written as:

(3) $$f\left(T,\tau ,\Theta \right)=\exp\left[-\frac{{\left(\Delta T\right)}^{2}}{2\sigma_{T}^{2}}-\frac{{\left(\Delta \tau \right)}^{2}}{2\sigma_{\tau }^{2}}\right]$$

#### 2.2.2. Gaussian + Skewness (GS)

To capture asymmetric decay behavior around the centering locations, the standard model is extended with a skew modulation mechanism.

(4) $$\Theta ={\left[\begin{array}{cccccc} \;T^{*} & \tau^{*} & \sigma_{T} & \sigma_{\tau } & \beta_{T} & \beta_{\tau }\; \end{array}\right]}^{T}$$

(5) $$f\left(T,\tau ,\Theta \right)=\exp\left[-\frac{{\left(\Delta T\right)}^{2}}{2{\left(\sigma_{T}e^{\beta_{T}\tanh\left(\Delta T\right)}\right)}^{2}}-\frac{{\left(\Delta \tau \right)}^{2}}{2{\left(\sigma_{\tau }e^{\beta_{\tau }\tanh\left(\Delta \tau \right)}\right)}^{2}}\right]$$

where $\beta_{T}$ and $\beta_{\tau }$ are skewness parameters of assistance timing and magnitude, respectively. These parameters enable asymmetric decay around the peak while preserving its position.

#### 2.2.3. Gaussian + Coupling (GC)

To capture potential interactions between timing and magnitude, a coupling term is introduced to allow cross-dimensional interaction between timing and magnitude.

(6) $$\Theta ={\left[\begin{array}{ccccc} \;T^{*} & \tau^{*} & \sigma_{T} & \sigma_{\tau } & \rho \; \end{array}\right]}^{T}$$

(7) $$f\left(T,\tau ,\Theta \right)=\exp\left[-\frac{{\left(\Delta T\right)}^{2}}{2\sigma_{T}^{2}}-\frac{{\left(\Delta \tau \right)}^{2}}{2\sigma_{\tau }^{2}}-\rho \Delta T\Delta \tau \right]$$

where ρ is the coupling parameter.

#### 2.2.4. Gaussian + Skewness + Coupling (GSC)

The full model incorporated both skew modulation and a coupling term.

(8) $$\Theta ={\left[\begin{array}{ccccccc} \;T^{*} & \tau^{*} & \sigma_{T} & \sigma_{\tau } & \beta_{T} & \beta_{\tau } & \rho \; \end{array}\right]}^{T}$$

(9) $$f\left(T,\tau ,\Theta \right)=\exp\left[-\frac{{\left(\Delta T\right)}^{2}}{2{\left(\sigma_{T}e^{\beta_{T}\tanh\left(\Delta T\right)}\right)}^{2}}-\frac{{\left(\Delta \tau \right)}^{2}}{2{\left(\sigma_{\tau }e^{\beta_{\tau }\tanh\left(\Delta \tau \right)}\right)}^{2}}-\rho \Delta T\Delta \tau \right]$$

Among the candidate surface models, a single model is selected for subsequent analysis. The selection is based on the trade-off between model accuracy, complexity, and cross-gait consistency. The final selection is reported in Section 4.1.

For each gait condition *g*, the corresponding scan experiment data are used in conjunction with the selected surface model to estimate the surface parameter vector Θ(g). Specifically, this estimation is performed by minimizing the reconstruction error between the measured physiological response and the model prediction, as shown in Equation (10). Then, the gait-specific assistance–response surface is constructed as Equation (11).

(10) $$\Theta \left(g\right)=\arg\min_{\Theta }\sum_{\left(T,\tau \right)}{∥{\tilde{J}}_{g}\left(T,\tau \right)-f\left(T,\tau ,\Theta \right)∥}_{2}^{2}$$

(11) $${\hat{J}}_{g}\left(T,\tau \right)=f\left(T,\tau ,\Theta \left(g\right)\right)$$

### 2.3. Gait–Surface Relationship Model

Based on the fitted surfaces under different gait conditions, the corresponding surface parameter sets are obtained. To model the dependence of the assistance–response relationship on gait conditions, we formulate a gait-conditioned modeling framework that maps gait variables to the parameter set, as depicted in Figure 1C.

A second-order bivariate polynomial basis expansion is adopted to represent the gait space, defined as:

(12) $$\psi \left(g\right)={\left[1,v,\phi ,v^{2},v\phi ,\phi^{2}\right]}^{T}$$

The gait–surface relationship is then modeled using a linear-in-parameters formulation based on the basis expansion in Equation (12). Each parameter $\theta_{k}\in \Theta$ is independently modeled as:

(13) $${\hat{\theta }}_{k}\left(g\right)=a_{k}^{T}\psi \left(g\right)$$

where ${\hat{\theta }}_{k}\left(g\right)$ denotes the estimated *k*-th parameter of the surface under gait condition *g*. $a_{k}\in R^{6\times 1}$ is the corresponding regression coefficient vector for the *k*-th parameter.

Stacking all parameters yields a compact matrix representation of the gait–surface relationship model:

(14) $$\hat{\Theta }\left(g\right)=A\;\psi \left(g\right)$$

where $\hat{\Theta }\left(g\right)\in R^{K\times 1}$ denotes the estimated surface parameter vector, $A\in R^{K\times 6}$ is the regression coefficient matrix, and *K* denotes the number of parameters in the surface model.

Given the estimated surface parameter sets obtained under all gait conditions in the scan dataset, we perform parameter identification to learn the regression coefficient matrix A, as expressed in Equation (15).

(15) $$A^{*}=\arg\min_{A}\sum_{i=1}^{N}{∥\Theta \left(g_{i}\right)-A\psi \left(g_{i}\right)∥}_{2}^{2}$$

where $A^{*}$ denotes the optimal regression coefficient matrix to be identified, and *N* is the number of modeled gait conditions.

### 2.4. Optimization Guidance

The application of the proposed model in optimization guidance is illustrated in Figure 1D. Based on the gait–surface relationship model Equation (14), given a new gait condition $g_{new}$, the surface parameter set can be estimated as:

(16) $$\hat{\Theta }\left(g_{new}\right)=A\;\psi \left(g_{new}\right)$$

Then, the assistance–response surface is constructed as:

(17) $${\hat{J}}_{g_{new}}\left(T,\tau \right)=f\left(T,\tau ,\hat{\Theta }\left(g_{new}\right)\right)$$

The structural information encoded in the surface can be further utilized to guide the configuration of the optimization process. For CMA-ES-based HIL optimization, the guidance includes the following aspects:

#### 2.4.1. Mean Initialization

In CMA-ES, the mean parameters $\mu_{t}$ and $\mu_{\tau }$ define the center of the sampling distribution for generating the initial population of candidate solutions [32]. In this study, their values are determined by the centering locations of the constructed surface:

(18) $$\mu_{0T}=T^{*},\;\mu_{0\tau }=\tau^{*}$$

#### 2.4.2. Scale Adaptation

The initial step-size parameter $\sigma_{0}$ controls the magnitude of stochastic perturbations around the mean in the first generation of sampling [32]. It is set to be proportional to the geometric mean of the surface scale parameters along both dimensions:

(19) $$\sigma_{0}=\kappa \sqrt{\sigma_{T}\cdot \sigma_{\tau }}$$

where κ is a scaling coefficient.

#### 2.4.3. Boundary Constraint

The boundary constraint defines the feasible search region of candidate solutions [32]. It is determined by the structure of the surface. Specifically, a high-confidence region is first extracted from the response surface:

(20) $$D=\{\left(T,\tau \right)\;|\;{\hat{J}}_{g}\left(T,\tau \right)\geq \alpha \}$$

where *D* denotes the high-confidence region, and α is a threshold that controls the range of the selected region.

The boundary of the search space is then defined by the extrema of each parameter dimension within *D*:

(21) $$T_{l}=\min_{(T,\tau )\in D}T,\;T_{u}=\max_{(T,\tau )\in D}T$$

(22) $$\tau_{l}=\min_{(T,\tau )\in D}\tau ,\;\tau_{u}=\max_{(T,\tau )\in D}\tau$$

where $T_{l},T_{u}$ are the lower and upper bounds of assistance timing, $\tau_{l},\tau_{u}$ are the bounds of magnitude, respectively.

The above guidance strategies incorporate the structural information of the predicted response surface into HIL optimization. By providing gait-dependent initialization and configurations, the proposed approach enables surface-informed exploration instead of a gait-independent generic search.

## 3. Experimental Method

### 3.1. Exoskeleton Platform

**Figure 2:**
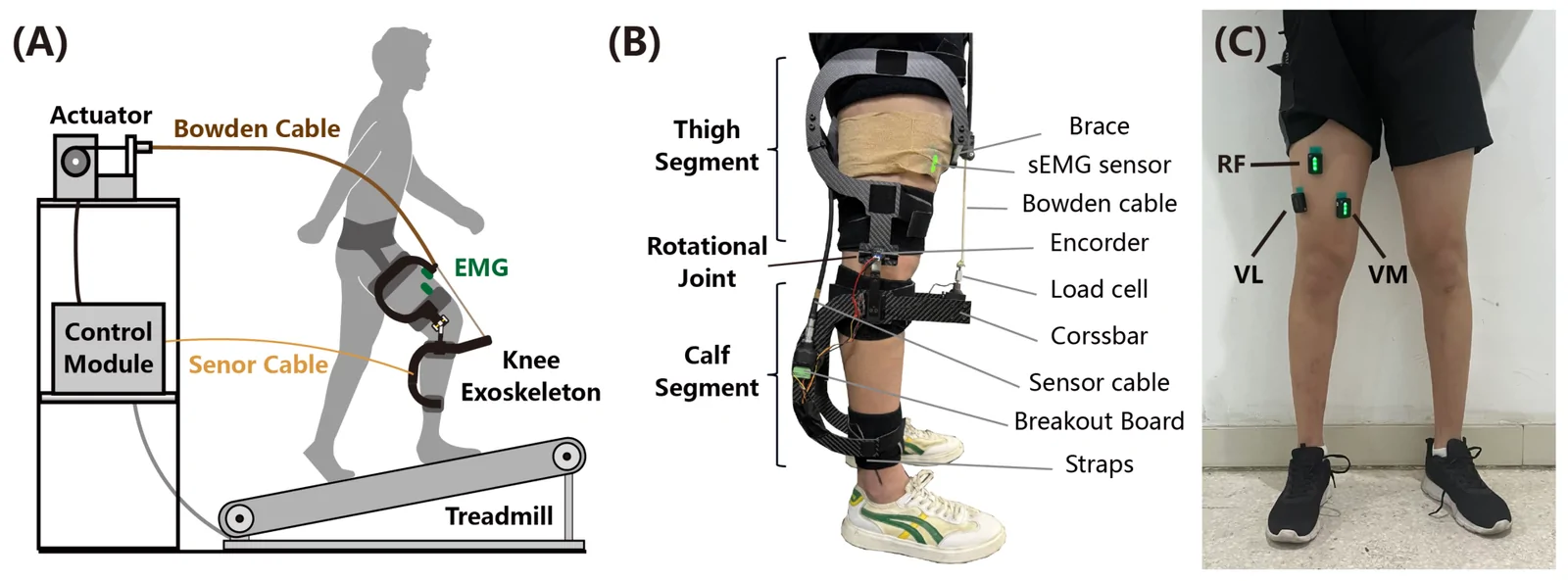
Experimental platform. (**A**) Overview of exoskeleton system. (**B**) Knee exoskeleton mechanical structure. (**C**) Placement locations of the wireless sEMG sensors.

The experimental platform used for this study consisted of a knee exoskeleton providing knee extension assistance, an off-board actuator unit with Bowden cable transmission, a variable-slope instrumented treadmill, a real-time control module, and a wireless surface electromyography (sEMG) system, as shown in Figure 2A.

The knee exoskeleton (Figure 2B) is composed of a thigh segment, a calf segment, and a rotational joint connecting the two segments. The Bowden cable connected the brace on the thigh segment to the crossbar on the calf segment, enabling knee extension actuation. A magnetic encoder (PQY18, ACCNT, Dongguan, China) was installed at the joint axis to measure joint angle. A load cell (DYMH-106, DAYSENSOR, Bengbu, China) was mounted on the crossbar to measure the assistance force or torque. Detailed mechanical design information of the exoskeleton was provided in [10].

The off-board actuator consisted of an AC brushless servo motor, a motor driver, and a 5:1 planetary gear system (BSM90N175AA, GBSM90 MRP120-5, and MF180-04AN-16A, ABB, Zurich, Switzerland), which drove the exoskeleton via a Bowden cable. A digital optical encoder (E5, US Digital, Vancouver, WA, USA) was used to measure the motor position. The treadmill (FIT, Bertec, Columbus, OH, USA) provided adjustable walking trials with varying speeds and slopes, and ground reaction force measurements were used for gait demarcation. A high-speed real-time controller (DS1202, dSPACE, Paderborn, Germany) was used to perform control tasks, sampling sensor data and calculating control commands at 500 Hz.

Wireless electrodes (Trigno, Delsys, Natick, MA, USA) were used to measure muscle activities with sEMG signals (see Figure 2C).

### 3.2. Assistance Formulation and Implementation

The exoskeleton assistance is realized by applying a knee extension torque to the human body. The desired torque profile is planned over the gait cycle and designed as a single-peaked curve, which has been shown to be effective in previous studies [1,2]. This profile is parameterized by four parameters, including peak time $T_{p}$, peak torque $\tau_{p}$, rise time $T_{r}$, and fall time $T_{f}$, as expressed in Equation (23).

(23) $$\tau^{des}\left(t\right)=\left\{\begin{array}{cc} \frac{1-\cos\left(\frac{t-T_{p}+T_{r}}{T_{r}}\pi \right)}{2}\tau_{p},\; & T_{p}-T_{r}\leq t<T_{p}\\ \frac{1+\cos\left(\frac{t-T_{p}}{T_{f}}\pi \right)}{2}\tau_{p}, & T_{p}\leq t<T_{p}+T_{f}\\ 0, & \mathrm{otherwise} \end{array}\right.$$

where $\tau^{des}$ is the desired torque, *t* represents the gait cycle.

Among the four parameters, peak time and peak torque are selected as the assistance timing *T* and assistance magnitude τ in the proposed model, while the rising and falling durations are fixed, as their influence on assistance effectiveness has been reported to be limited in previous studies [15,30]. The fixed values are set to $T_{r}=10\%$ and $T_{f}=10\%$, respectively, following prior work [10,33].

To ensure accurate tracking of the desired torque profile, the cable-tightness-based controller (CTBC, detailed in [33]) is adopted due to its enhanced robustness under parameter-varying assistance.

### 3.3. Physiological Response Function

The physiological effect of exoskeleton assistance is evaluated based on the activation of three major knee extensors on the assisted side: rectus femoris (RF), vastus medialis (VM), and vastus lateralis (VL), because reductions in their activation indicate reduced muscular demand associated with knee extension assistance [4,10,34]. This selection is consistent with previous studies [29]. Muscle activities are quantified using sEMG signals. The placement locations of the wireless sEMG sensors are shown in Figure 2C.

The raw sEMG data were sampled at 500 Hz and processed in three stages [35]. First, a second-order high-pass Butterworth filter with a cutoff frequency of 20 Hz was applied to remove motion artifacts. Second, the signals were full-wave rectified. Third, a second-order low-pass Butterworth filter with a cutoff frequency of 10 Hz was used to generate linear envelopes of muscle activation.

The physiological response function is defined as the unweighted average of the step-averaged RMS values of filtered and normalized sEMG signals from the three target muscles, as expressed in Equation (24). The formulation of this function is based on previous studies [15,29].

(24) $$J=\frac{1}{3n}\sum_{i=1}^{n}\sum_{m\in \{RF,VL,VM\}}\;\sqrt{\sum_{t_{i}=0}^{T_{Di}}\frac{1}{T_{Di}}{\left(\frac{EMG_{m}\left(t_{i}\right)}{EMG_{m}^{norm}}\right)}^{2}}$$

where *i* and *m* are the indices of steps and muscles, *n* is the total number of tested steps, $t_{i}$ and $T_{Di}$ are the current time and total duration of step *i*, $EMG_{m}$ is the sEMG linear envelope of muscle *m*, and $EMG_{m}^{norm}$ is the normalization factor of muscle *m*, which was obtained by measuring the step-averaged RMS during 2-min unassisted walking.

### 3.4. Human-in-the-Loop Optimization

The HIL optimization procedures in this study were based on a CMA-ES framework [1]. The assistance parameters $T_{p}$ and $\tau_{p}$ were optimized to minimize the cost function Equation (24). At each iteration, a population of candidate assistance parameter sets was generated from a multivariate normal distribution, defined by mean, standard deviation, boundary, and covariance matrix. The number of candidate patterns included in each generation was set to six [32]. Each pattern was applied for 20 steps to evaluate the corresponding physiological response function, and the first five steps were excluded as transitional steps. Then, the performance rankings of these patterns were used to update the distribution parameters for the next generation; one generation of online HIL optimization generally required approximately 3–5 min. This iterative process was repeated from one generation to the next. After a number of generations of evaluation, the mean of the subsequent generation is taken as the current estimate of the optimal parameter values. Previous HIL studies [15,30,36] on two- or four-parameter exoskeleton assistance optimization commonly used four generations to achieve effective assistance performance.

### 3.5. Participants

Five participants (aged 23.8±1.3 years; height 177.4±4.6 cm; body mass 74.4±6.3 kg) were recruited for the experiments. The individual participant characteristics are listed in Table 1. All participants were healthy individuals without any movement disorders or discomfort when wearing the exoskeleton. Participant 1 was involved in all scan experiments of the dataset construction study, while Participants 1–5 each took part in one gait condition of the validation study. All participants provided written informed consent before completing the experiments. The study was approved by the ethical committee of Nankai University (NKUIRB2025084).

### 3.6. Dataset Construction Study

To construct a comprehensive dataset for modeling the gait–assistance–response relationship, systematic physiological response sampling was performed across selected gait conditions and assistance parameter configurations.

**Figure 3:**
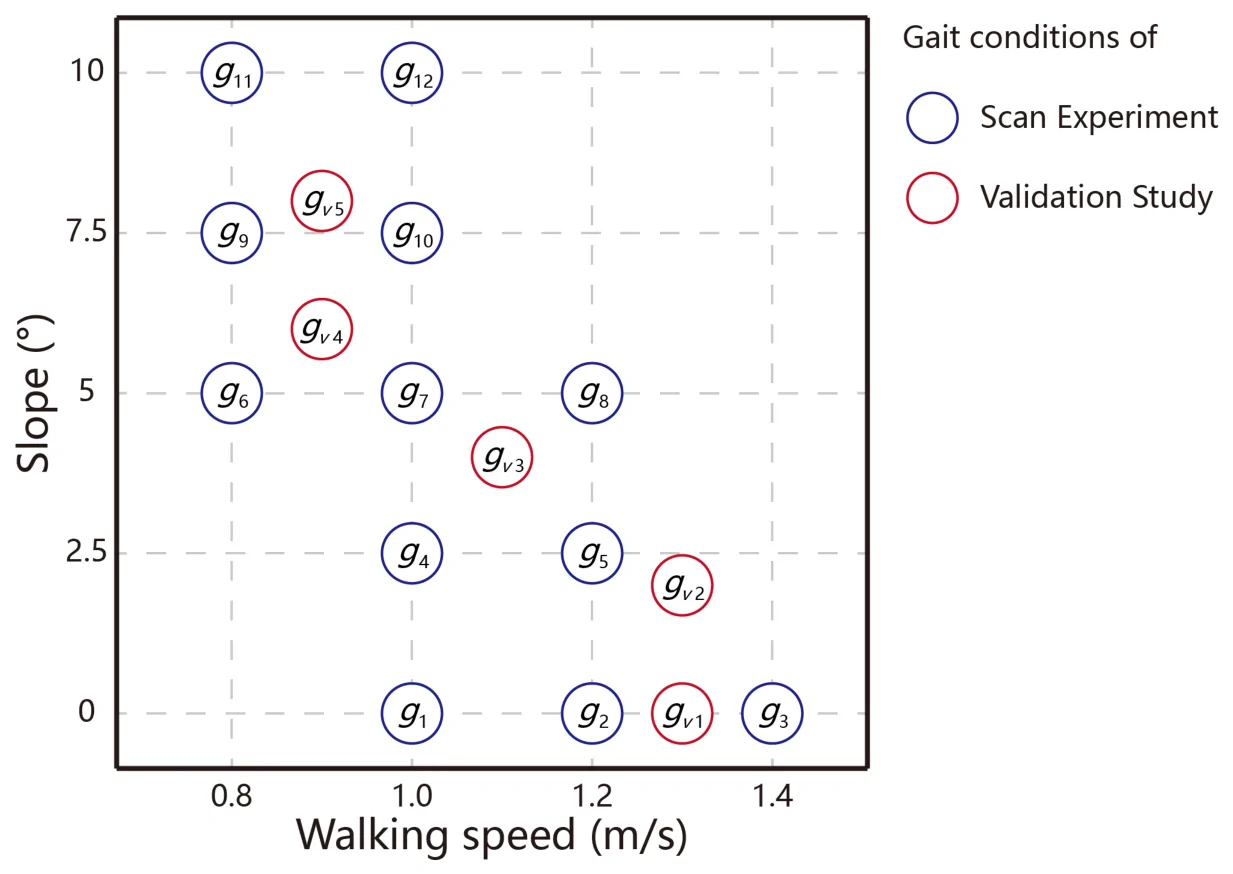
The selected gait conditions of the scan experiments and validation study. The circle locations represent gait parameters in the speed–slope space, while the labels indicate the index of each condition.

Twelve gait conditions covering different walking speeds and slopes were selected, as shown in Figure 3. For each gait condition, a 2-min unassisted walking period was first conducted to allow participant adaptation and compute sEMG normalization factors. Subsequently, a scan experiment over the assistance parameter space was conducted, using an identical 6 × 6 parameter grid across all gait conditions. Specifically, the grid consisted of six peak timing levels ($T_{p}\in \left\{10\%,12\%,14\%,16\%,18\%,20\%\right\}$ gait cycle) and six peak torque levels ($\tau_{p}\in \left\{0.20,0.24,0.28,0.32,0.36,0.40\right\}$ Nm/kg body mass). The 36 assistance patterns were applied following a predefined traversal order across the grid, with torque levels prioritized in ascending order, followed by variations along the time-delay dimension within each torque level. Each assistance pattern was maintained for 15 consecutive steps in the forward direction and then repeated in a reverse direction for another 15 steps. This bidirectional execution protocol was designed to mitigate the effects of physiological drift and fatigue. After each assistance-pattern change, the first five steps in each 15-step evaluation window were excluded as transitional steps, and the response value was calculated using the remaining 10 steps. In this way, each assistance pattern $\left(T_{p},\tau_{p}\right)$ yielded n=20 steps of muscle-activation data under the current gait condition *g*, which were then used to compute the corresponding physiological response function $J_{g}\left(T_{p},\tau_{p}\right)$ according to Equation (24).

In summary, the scan experiments yielded physiological response function data across 12 gait conditions and 36 assistance patterns. These data were subsequently used for 12 gait-specific surface fittings and one global regression, enabling the construction of the hierarchical relationship model.

### 3.7. Validation Study

To provide a preliminary evaluation of the capability of the proposed framework in predicting effective assistance patterns and improving optimization efficiency across different conditions, validation experiments were conducted under five gait conditions distinct from those used in the scan experiments. The five validation gait conditions were defined as follows: $g_{v1}$ = (1.3 m/s, 0°), $g_{v2}$ = (1.3 m/s, 2°), $g_{v3}$ = (1.1 m/s, 4°), $g_{v4}$ = (0.9 m/s, 6°) and $g_{v5}$ = (0.9 m/s, 8°), as illustrated in Figure 3.

**Figure 4:**
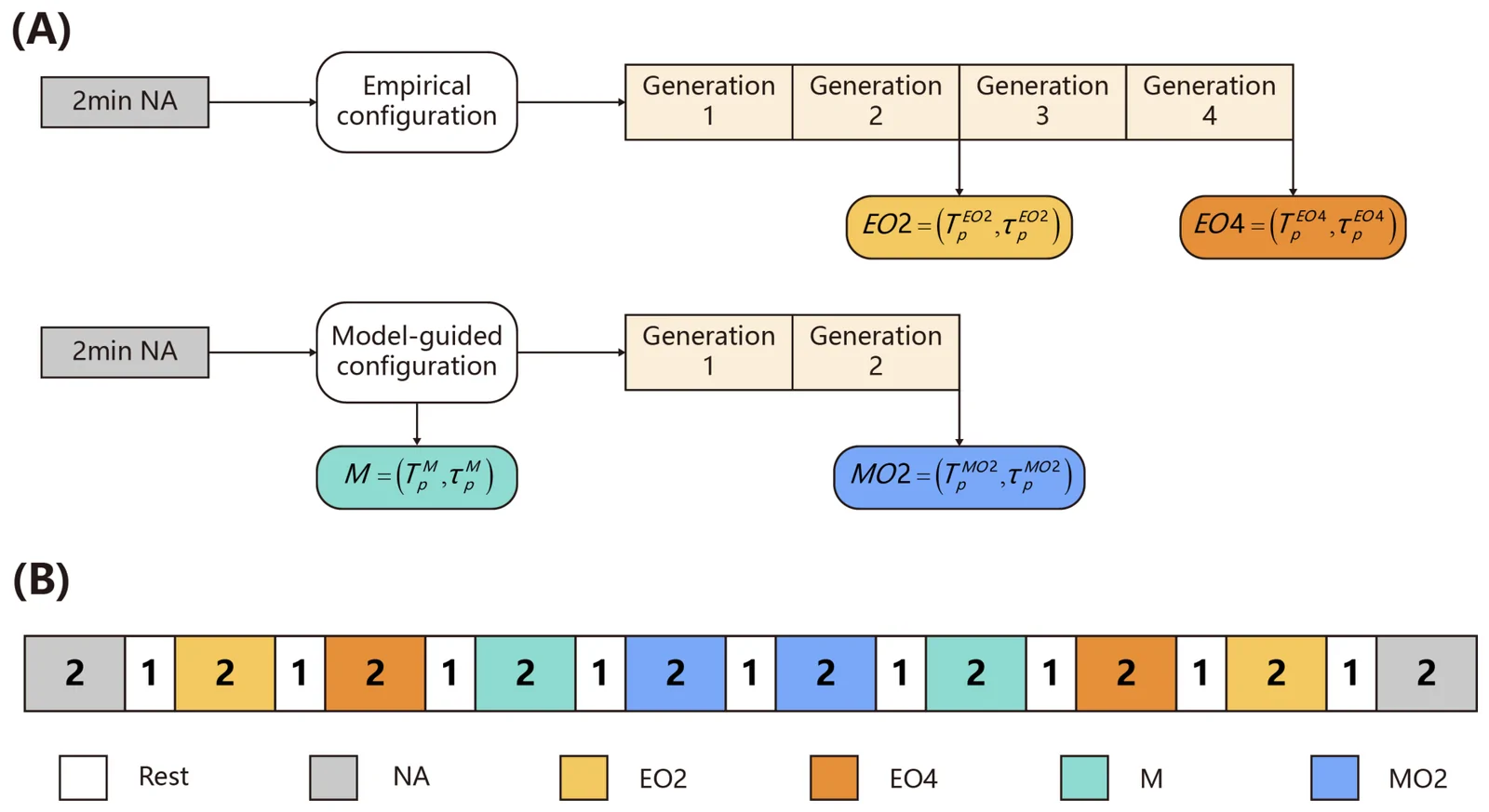
Experimental protocol of each validation gait condition. (**A**) Four-generation empirical HIL optimization produced patterns EO2 and EO4, model prediction yielded *M*, two-generation model-guided HIL optimization produced the pattern MO2. (**B**) Procedure of the comparative experiment. The colored blocks represent the experimental steps, and the numbers indicate the duration (min) of each process.

Each participant performed one validation gait condition. Specifically, Participant 1 performed condition $g_{v5}$, whereas Participants 2–5 performed conditions $g_{v1}$–$g_{v4}$, respectively. The proposed strategy was therefore evaluated under participant–gait-condition combinations. Three experimental protocols were subsequently conducted for each condition in sequence: an empirical HIL optimization, a model-guided HIL optimization, and a comparative experiment (see Figure 4), with an interval of at least one day between protocols.

The configuration of the empirical HIL optimization was determined based on prior studies [10,15], and empirical experience. Specifically, the initial mean was set to $T_{p}=15\%$ gait cycle and $\tau_{p}=0.3$ Nm/kg; the initial standard deviation was set to 0.2; the search space was constrained to $T_{p}\in \left[10\%,20\%\right]$ gait cycle and $\tau_{p}\in \left[0.18,0.45\right]$ Nm/kg. The optimization procedure was performed for four generations preceded by a 2-min no-assistance walking (Figure 4A), the optimized patterns after two-generation empirical HIL optimization and four-generation empirical HIL optimization were recorded as $EO2=(T_{p}^{EO2},\tau_{p}^{EO2})$ and $EO4=(T_{p}^{EO4},\tau_{p}^{EO4})$, respectively. The configuration of the model-guided HIL optimization was determined using Equations (18), (19), (21) and (22). The optimization procedure was performed for two generations, model-predicted initial mean and the optimized pattern after two-generation model-guided HIL optimization were denoted as $M=(T_{p}^{M},\tau_{p}^{M})$ and $MO2=(T_{p}^{MO2},\tau_{p}^{MO2})$ respectively.

The comparative experiment consisted of five conditions: no-assistance walking (NA) and four assistance patterns (EO2, EO4, *M*, and MO2). Each condition consisted of two 2-min trials, one performed in the forward order and the other in the reverse order. A 1-min rest interval was provided between adjacent trials. The protocol is illustrated in Figure 4B. For each condition, only the last 30 steady-state steps from each trial were used for analysis. The final condition-wise results were obtained by averaging the results of the two trials.

## 4. Results

### 4.1. Scan Results and Surface Reconstruction

**Figure 5:**
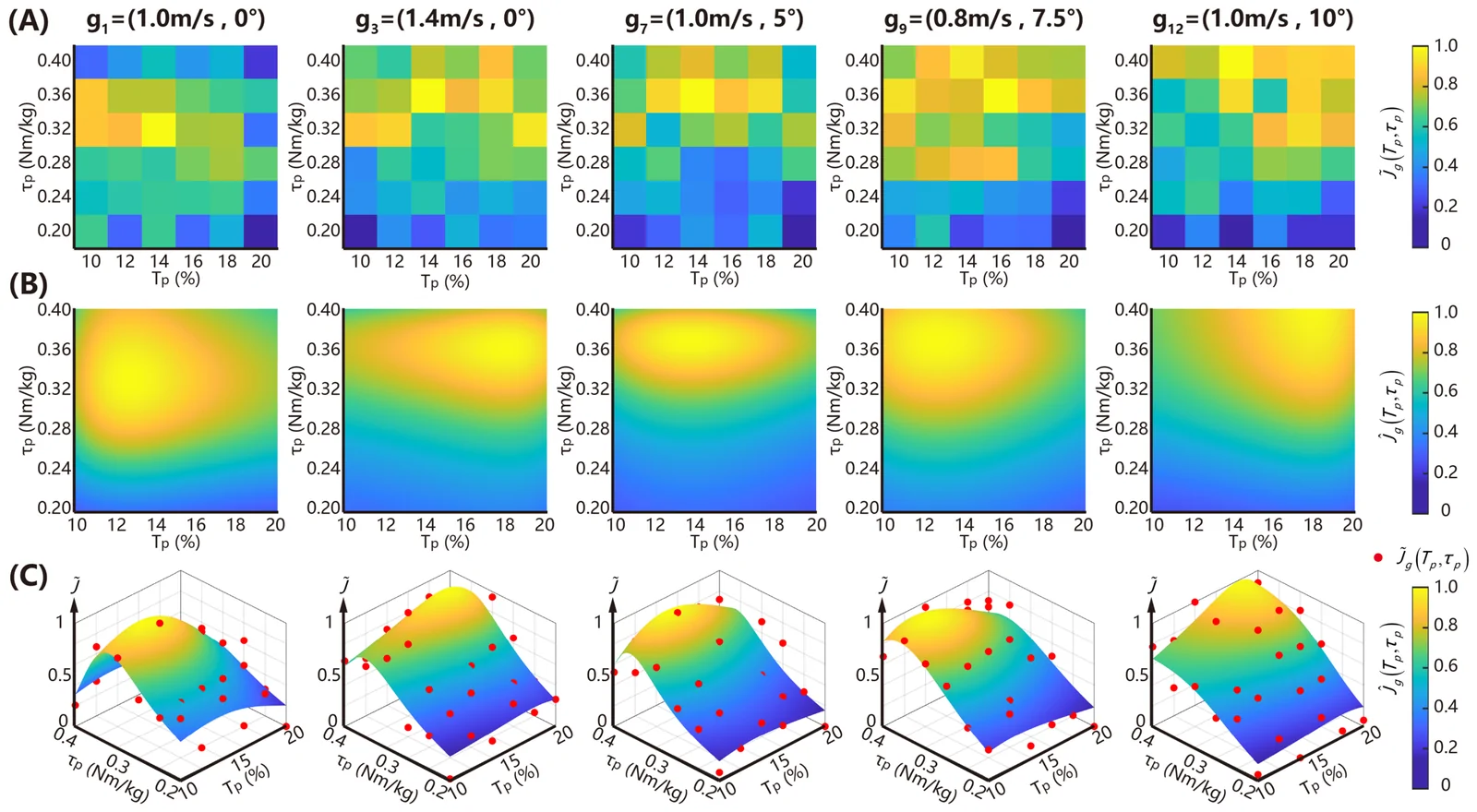
(**A**) Scan Results. Each panel corresponds to a specific gait condition, and each grid cell represents an evaluated assistance pattern. The color indicates the normalized physiological response function. (**B**) Heatmap representation of the fitted response surface, where the color encodes the surface value. (**C**) Reconstructed response surface with overlaid scan data points (marked by red dots).

The scanning experiments collected muscle-activation data from different gait conditions and assistance patterns. Given that the response function Equation (24) is inversely correlated with assistance effectiveness, an inverse min–max normalization was performed within each gait condition to obtain the normalized response ${\tilde{J}}_{g}\left(T_{p},\tau_{p}\right)$. Several representative gait conditions were selected for visualization in Figure 5A.

For each gait condition, the candidate surface models in Equations (3), (5), (7) and (9) were independently fitted to the scan data. The scan range parameters in Equation (2) were determined according to the experimental design, with $T_{\min}=10\%$, $T_{\max}=20\%$, $\tau_{\min}=0.20$ Nm/kg, and $\tau_{\max}=0.40$ Nm/kg. The coefficient of determination ($R^{2}$), root mean squared error (RMSE) and mean absolute error (MAE) were used to evaluate the model performance in terms of goodness of fit and reconstruction error. The average values of the three indicators across all gait conditions are reported in Table 2.

The performance of the four candidate models followed the ranking GSC > GS > GC > G. Paired *t*-tests were conducted on the $R^{2}$ values across gait conditions, and all comparisons were found to be statistically significant (p<0.05). However, the improvement of GSC over GS was marginal (0.008 in $R^{2}$), despite the introduction of an additional parameter that increased model complexity and may affect the stability of subsequent regression. Therefore, GS was selected as the final surface representation for subsequent modeling and analysis.

The fitted surfaces derived from Model GS for representative gait conditions are visualized as two-dimensional projections in Figure 5B, while the corresponding three-dimensional surfaces with the original scan data points overlaid for comparison are shown in Figure 5C.

### 4.2. Gait-to-Surface Regression Analysis

The structural parameters of the fitted surfaces were extracted to analyze their dependence on gait conditions defined by walking speed and slope.

**Figure 6:**
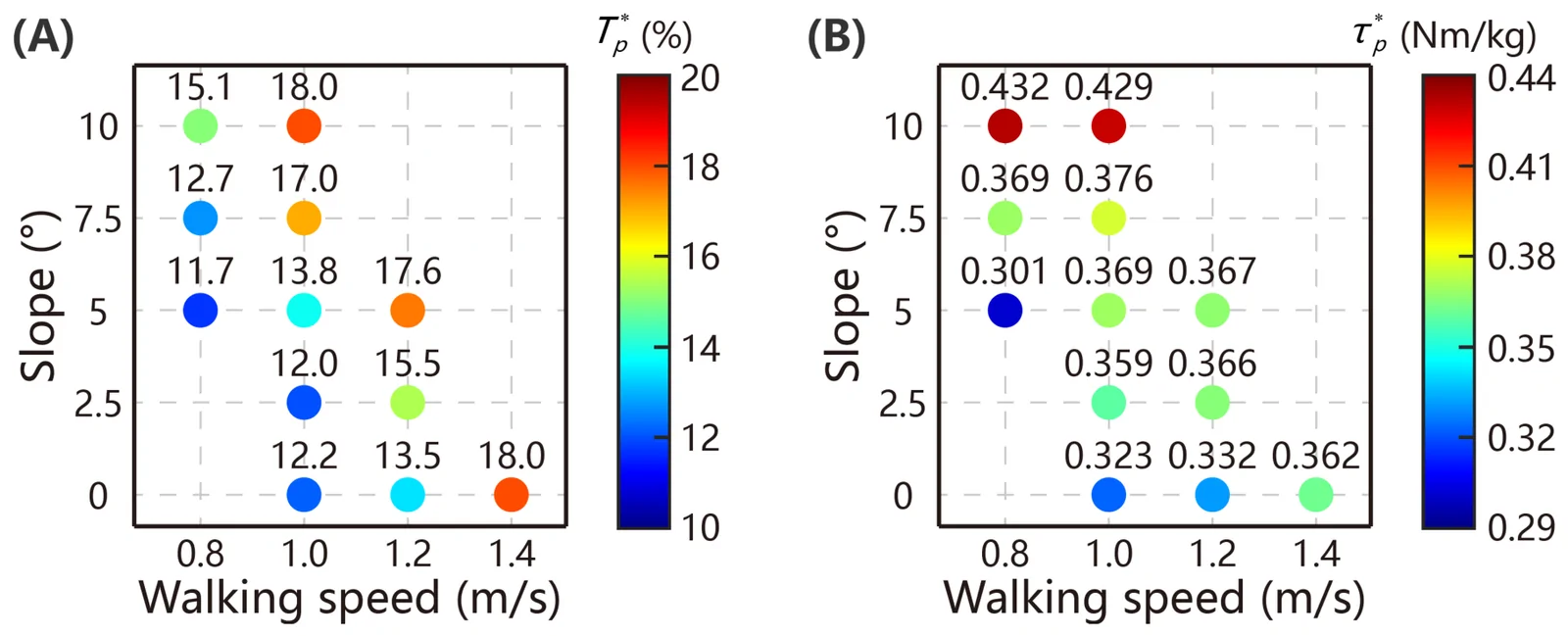
The distributions of (**A**) the centering location of peak time and (**B**) the centering location of peak torque in the gait space. The color represents the parameter magnitude.

The distributions of the centering location of timing and magnitude in the gait space are visualized in Figure 6. A consistent trend can be observed, where higher walking speed or steeper slope tends to shift the timing center toward later phases, with walking speed showing a relatively stronger influence. In addition, increases in both walking speed and slope are generally associated with larger magnitude values, while slope appears to exhibit a comparatively greater influence on magnitude than walking speed.

To quantitatively model the relationship between gait-condition variables and surface parameters, the second-order bivariate regression introduced in Equations (14) and (15) was performed using the 12 gait-surface pairs as independent samples. The solution was obtained via least-squares estimation. The regression coefficients are summarized in Table 3, and the regression analysis results in terms of the $R^{2}$ for the six parameters are reported in Table 4.

To further evaluate the prediction error of the high-level model under held-out gait conditions, a leave-one-gait-condition-out cross-validation (LOOCV) analysis was conducted. In each fold, one gait condition was held out, and the model fitted from the remaining 11 conditions was used to predict its surface parameters. MAE, RMSE, and normalized MAE were used to quantify the prediction errors. The normalized MAE was calculated by dividing the MAE by the range of the corresponding surface parameter across the 12 scan gait conditions. The results are reported in Table 4.

Most structural parameters exhibited high $R^{2}$ values, indicating that the regression model effectively captured their dependence on gait conditions. The LOOCV results further provided an estimate of the prediction errors under held-out gait conditions. The center parameters $T_{p}^{*}$ and $\tau_{p}^{*}$ showed relatively moderate normalized MAE values of 12.98% and 17.01%, respectively. In comparison, $\sigma_{Tp}$ showed a relatively lower $R^{2}$ value (0.435) and a higher normalized MAE (33.45%). A relatively higher normalized MAE was also observed for $\beta_{\tau p}$ (28.13%).

### 4.3. Candidate Assistance Patterns

**Figure 7:**
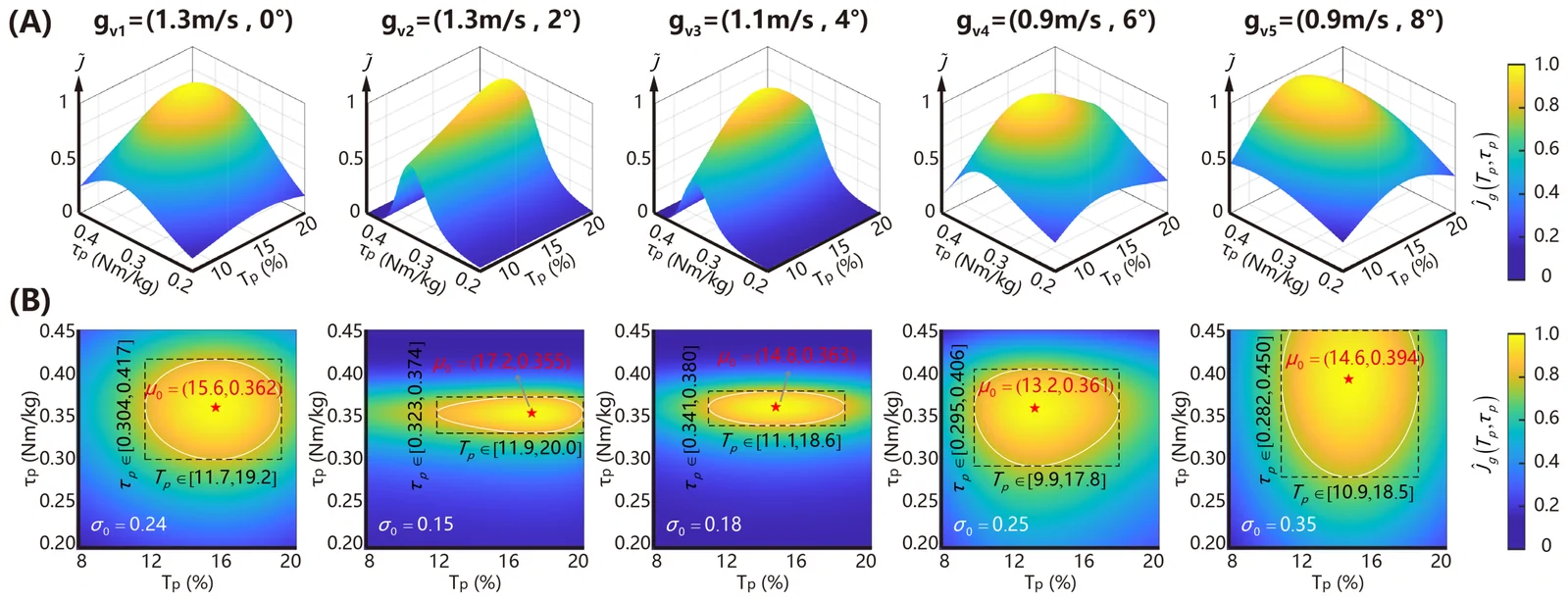
(**A**) Model predicted response surfaces under five validation gait conditions. (**B**) Surface-guided optimization configurations. The background color represents the 2D projection of the response surface in the assistance parameter space. The red star indicates the centering locations of the assistance parameters, the white contour denotes the high-confidence region, and the black dashed boundary defines the feasible parameter range. The corresponding optimized configuration parameters are annotated.

Using the coefficient matrix obtained from Table 3, the surface parameters under the validation gait conditions ($g_{v1}$–$g_{v5}$) were estimated according to Equation (16). Based on these estimated parameters, the response surfaces were reconstructed, as illustrated in Figure 7A.

The reconstructed surfaces and their corresponding parameters were then used to guide the optimization configuration. The high-confidence region was defined using a threshold α=0.8, and the scaling coefficient was set to κ=0.5, which empirically balanced exploration and exploitation in the optimization process. Additionally, a hard constraint $\tau_{p}\leq 0.45$ Nm/kg was imposed to ensure safety. The resulting optimization setups are illustrated in Figure 7B.

By conducting the optimization experiments described in Section 3.7, candidate assistance patterns under each validation condition were obtained, as shown in Table 5.

### 4.4. Validation Results

**Figure 8:**
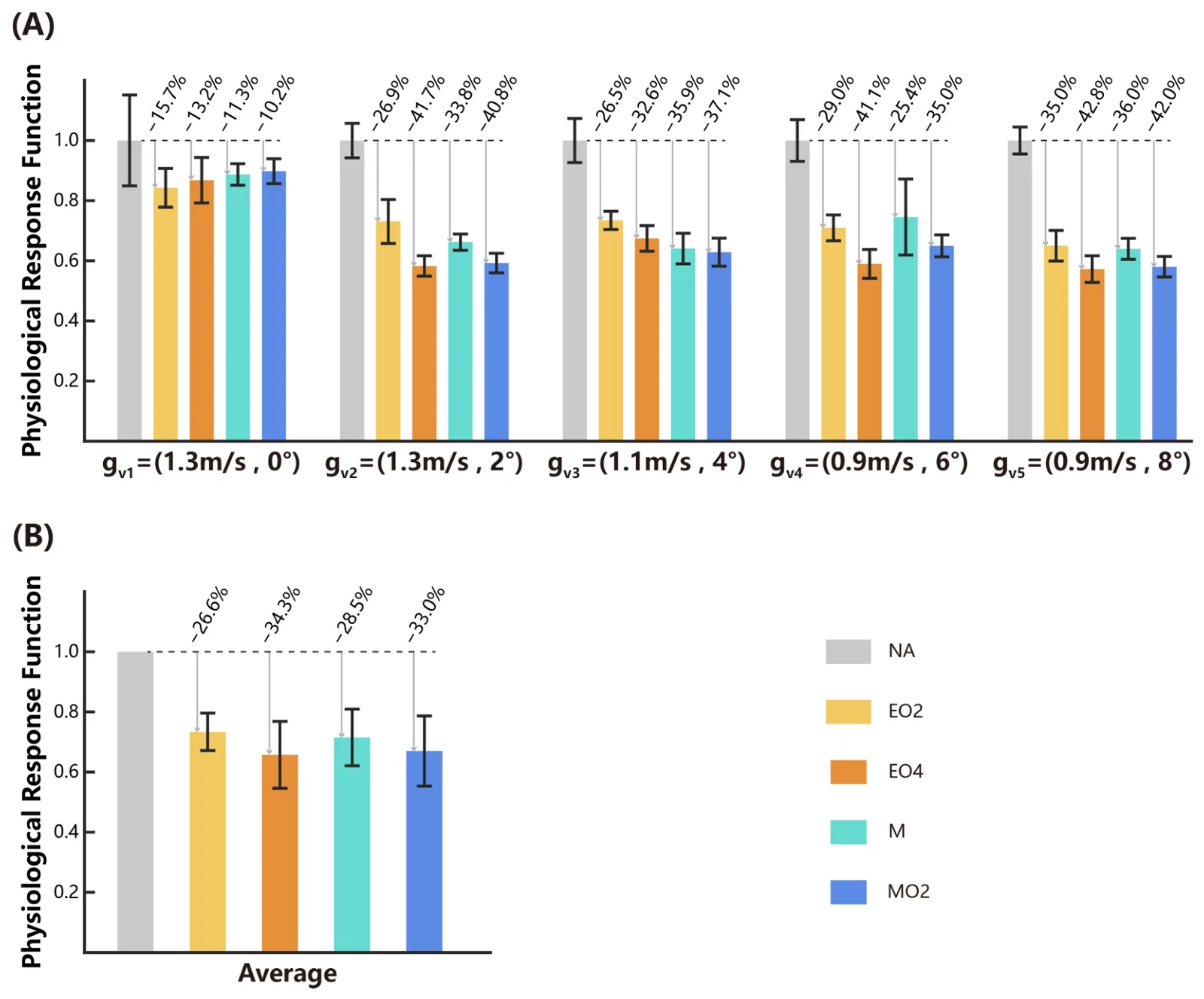
Physiological response function comparison across assistance patterns obtained under different generation strategies. (**A**) Results under each validation gait condition, normalized using the no-assist condition as baseline. Each validation gait condition was performed by one participant. Error bars indicate inter-step standard deviation within each condition. (**B**) Averaged results across validation conditions, where error bars represent standard deviation across validation conditions.

In the comparison experiments under five validation gait conditions, all candidate assistance patterns consistently reduced the activation level of the knee extensor muscles. To ensure comparability across different conditions, the physiological response function of each pattern was normalized with respect to the corresponding no-assistance baseline. The normalized results are presented in Figure 8.

In terms of the average performance across all validation conditions, EO2, EO4, *M*, and MO2 reduced muscle activation by 26.6%, 34.3%, 28.5%, and 33.0%, respectively, compared with the no-assistance condition. The model-only strategy (*M*), which did not require iterative optimization, achieved a slightly higher average reduction than EO2, although this trend was not consistent across all individual validation conditions. Furthermore, MO2, which used the same optimization budget as EO2, showed a mean additional 6.4 percentage-point reduction in muscle activation on average, corresponding to a 24.1% relative improvement over EO2. It also closely approached EO4, with only a 1.3 percentage-point performance gap (or a 3.8% relative gap) despite requiring only half of the optimization iterations.

To assess whether the overall trend was driven by the inclusion of the model-development participant, a sensitivity analysis was performed by excluding Participant 1 and recalculating the average performance using only $g_{v1}$–$g_{v4}$. Across these four independent-participant validation conditions, the average reductions in muscle activation were 24.5%, 32.2%, 26.6%, and 30.8% for EO2, EO4, *M*, and MO2, respectively. The trend remained generally consistent with the full validation set. Specifically, MO2 showed a 6.3 percentage-point greater mean reduction than EO2 and a 1.4 percentage-point smaller mean reduction than EO4.

Paired *t*-tests based on the five validation conditions were conducted between the no-assistance condition and each of the four assistance strategies. All assistance strategies significantly reduced muscle activation compared with the no-assistance condition (EO2: *p* = 0.0010; EO4: *p* = 0.0035; *M*: *p* = 0.0038; MO2: *p* = 0.0048). The mean paired differences, 95% confidence intervals, and *p*-values for pairwise comparisons among the four assistance strategies are reported in Table 6.

Across the $g_{v2}$–$g_{v5}$ conditions, MO2 consistently outperformed *M* and EO2, while EO4 also showed stable superiority over EO2. However, an opposite trend was observed in $g_{v1}$, where all assistance strategies yielded only marginal reductions in muscle activation, with no clear performance separation among methods.

## 5. Discussion

This study aims to develop a gait-conditioned optimization framework for exoskeleton assistance, with the goal of improving the efficiency of HIL optimization across various gait conditions. A knee exoskeleton with extension assistance was selected as a case study, where the model was constructed and preliminarily evaluated based on muscle-activation reduction under five validation conditions. Although the framework was demonstrated in a specific application, it holds the potential to be extended to other exoskeleton devices (e.g., ankle [1,15], knee flexion [37], and hip exoskeletons [25,38]), optimization objectives (e.g., measured [2,28] or estimated [39] metabolic cost, gait quality [40,41]), and task scenarios (e.g., running [36], uphill/downhill walking [20,29], and upstairs/downstairs [38,42]).

The proposed strategy enables data-driven optimization conditioned on gait conditions. Conventional HIL optimization typically applies generalized optimization configurations across different gait conditions [26], requiring additional trials to evaluate and eliminate ineffective assistance patterns. Data-driven relationship models can leverage prior knowledge to constrain the search within high-confidence regions and reduce unnecessary exploration [30], thereby using prior model-development trials to reduce the online optimization cost across diverse gait conditions.

The hierarchical structure enables more comprehensive utilization of the collected data. Compared with a single-level relationship model mapping gait conditions to optimized assistance patterns [30], the proposed hierarchical model replaces the single optimized pattern with an assistance–response surface as the gait-conditioned prediction. The point-based estimation retains only the optimized assistance pattern for each gait condition, while the physiological responses associated with other tested patterns are not explicitly considered, although they may contain additional information about the underlying response landscape. By preserving this information, the surface-based representation may reduce the reliance on potentially noisy optimal solutions and can provide additional information for configuring optimization, including feasible ranges and exploration scales.

The primary use of the proposed model is to guide HIL optimization rather than to predict a deployable effective pattern. Although the direct output of the proposed model, *M*, can be regarded as a gait-dependent generalized assistance pattern, further personalized optimization remains necessary due to inter-individual variability [2,21]. In this sense, the model development focuses on identifying how effective assistance patterns vary with gait conditions, while user-specific adaptation is still provided by the subsequent HIL optimization process. Thus, for *M*, a reliably effective assistance pattern is preferred over a highly optimized but potentially sensitive solution. For this purpose, the proposed model was established based on scan experiments rather than optimization experiments, since systematic scans provide broader and more structured coverage of the assistance parameter space.

Surface models should aim to capture the underlying response landscape rather than precisely fit individual measurements. Due to inherent measurement noise and stochastic variability in physiological responses, the scan results across the assistance parameter space often exhibit local irregularities and fluctuations (see Figure 5A). Nevertheless, the fitting performance of the surface models (see Table 2) suggests that the assistance–response relationship exhibits a certain degree of consistent and learnable structure. Complex multi-modal models may be more flexible in capturing local variations but may compromise consistency across different gait conditions. Therefore, based on the approximately peak-centered and gradually decreasing trend of the muscle-activation benefit (see Figure 5A), we adopted simple and smooth convex surfaces to represent this underlying landscape. Although the predicted center of the surface may not coincide with the experimentally observed optimum, it is determined by the overall data distribution and therefore has a higher likelihood of representing an effective assistance region. Such a robust representation is more suitable for providing guidance for optimization.

The selection of the surface model depends on the specific exoskeleton system and objective function. In this study, the GS model was selected based on the scan results, as it provided a favorable balance between fitting accuracy and model complexity. However, other candidate models may remain applicable in other scenarios where additional response characteristics need to be captured. For example, when coupling effects are prominent, the coupling parameter ρ in GC and GSC may provide additional information for initializing the covariance structure of the HIL optimization.

Second-order polynomial regression was used to describe the relationship between gait parameters and surface parameters because it provided a favorable fitting performance in the collected experimental dataset while maintaining a relatively simple model structure. In the regression analysis of gait–surface relationships, most surface parameters achieved high regression performance, supporting the presence of systematic variations in surface characteristics across the current set of gait conditions. The LOOCV analysis further showed that the center parameters $T_{p}^{*}$ and $\tau_{p}^{*}$ had relatively moderate prediction errors under held-out gait conditions. The relatively low regression performance observed for $\sigma_{Tp}$ and the relatively higher held-out prediction error observed for $\sigma_{Tp}$ and $\beta_{\tau p}$ may be related to their reduced sensitivity with respect to the selected gait basis functions in Equation (12). These uncertainties may affect the predicted search-scale or surface-shape configurations, rather than directly determining the final assistance pattern. This suggests that a universal basis function set may not be optimal for all surface parameters, and future work may develop parameter-specific basis functions according to their individual distribution characteristics.

The proposed model also provides scalability for future expansion. In the current study, the high-level model was established by fitting the relationship between gait conditions and surface parameters using 12 gait–surface pairs. Future work can incorporate additional scan experiments from more gait conditions and participants into the model database, thereby further improving the reliability and generalizability of the learned relationships.

The guidance parameters, including the confidence threshold α and scaling coefficient κ, were empirically determined in this study to balance exploration and exploitation, although their sensitivity requires further systematic evaluation. Since these parameters may influence the search region and initial search scale, future work will further investigate their sensitivity and optimize their settings for different conditions.

The applicability of the proposed model is limited to the range covered by the scan experiments. Since both gait conditions and assistance parameters were explored within predefined ranges, the fitted and predicted response surfaces are expected to be reliable mainly within or near these regions. Therefore, extrapolation to substantially different gait conditions or assistance patterns beyond the experimental boundaries should be interpreted with caution.

In terms of control strategy, a novel controller [33] was employed instead of the iterative learning-based controllers [43] commonly used in related studies. This choice was motivated by the frequent variations in assistance parameters during the scan and optimization experiments, where a controller capable of handling parameter-varying conditions is preferred.

In the scan experiment, a forward and reverse evaluation procedure was used to reduce the influence of fatigue and physiological drift. The order of the 36 assistance patterns was fixed under each gait condition, which may still introduce potential systematic order effects. Future work will use randomized pattern orders to further reduce these effects.

In the validation experiments, the pairwise comparisons among the four assistance strategies did not reach statistical significance. This result may be related to the limited number of validation conditions and the variability across gait-condition–participant combinations. Specifically, the results under $g_{v2}$–$g_{v5}$ exhibited generally consistent trends, whereas $g_{v1}$ showed an opposite tendency with relatively smaller differences among optimization strategies. This discrepancy may be attributed to the lower demand for knee extension assistance during level-ground walking [10], where muscle-activation may be less sensitive to variations in assistance patterns. Nevertheless, participant-specific responses may also have contributed to this observation. Such condition-specific variability may have increased the variability of the paired differences, thereby reducing the statistical power of the pairwise comparisons among assistance strategies.

The participant involved in model construction performed validation condition $g_{v5}$. Under this condition, EO4 and MO2 showed similar assistance effects and both outperformed EO2 and *M*, whose effects were relatively closer to each other. This trend was generally consistent with the average results across all validation conditions. This observation suggests that the inclusion of the model-construction participant in validation did not produce a trend inconsistent with the overall results.

The comparison with previous studies further highlights the value of assistance customization. Under comparable gait conditions, all patterns evaluated in this study (EO2, EO4, *M* and MO2) showed greater reductions in muscle activation than the results reported for the generic assistance strategy in previous works, which employed the same [10] or similar [44] exoskeleton system. This suggests that customized patterns, either through optimization, gait-conditioned prediction, or their combination, may facilitate greater muscle-activation benefits than generalized patterns.

This study still has several limitations. First, the model-development dataset was collected from a single participant, which may limit the ability of the model to capture inter-participant variability in physiological responses. Second, the inclusion of only five participants in the validation experiment may limit the statistical strength of the conclusions. Third, in the validation experiment, each participant completed only one validation gait condition. Therefore, participant identity and gait condition were confounded, making it difficult to separately distinguish the effects of gait-condition variation and individual variability on the validation results. Accordingly, the modeling and validation results of this study should be interpreted as a preliminary proof-of-concept demonstration of the proposed model-guided HIL optimization strategy, rather than as the development of a final high-performance model or sufficient evidence of cross-user, multi-gait, or population-level generalizability. Future studies will construct models using data from more participants, more optimization objectives, and more gait conditions, and will further evaluate their cross-user and cross-gait-condition generalizability through larger-scale validation experiments.

## 6. Conclusions

This study developed a gait-conditioned optimization framework based on a gait–assistance–response relationship model to provide preliminary support for improving the efficiency of HIL optimization across different gait conditions. To this end, parameterized surfaces were proposed to characterize assistance–response landscapes under individual gait conditions, and global regression was used to establish gait–surface relationships for guiding HIL optimization configurations. Experiments were conducted on a knee exoskeleton with extension assistance, where the model was established using predefined gait conditions and used to guide HIL optimization under validation gait conditions. Compared with two-generation empirical HIL optimization, model-guided HIL optimization showed a mean additional 6.4 percentage-point reduction in muscle activation under the same number of optimization generations (6.3 percentage points after excluding the model-development participant). Moreover, it approximately approached the mean performance of four-generation empirical HIL optimization while requiring half of the optimization generations. The current experiments provide preliminary evidence that the proposed model-guided strategy can reduce the number of optimization generations while maintaining comparable assistance performance under the tested gait–participant combinations, and indicate its potential for improving multi-gait HIL optimization efficiency and supporting more efficient customization of effective assistance patterns. 

## Figures and Tables

**Table 1:** Individual participant characteristics.

Participant	Age (Years)	Height (cm)	Body Mass (kg)	Sex
Participant 1	25	180	71	Male
Participant 2	24	183	84	Male
Participant 3	25	178	72	Male
Participant 4	22	175	77	Male
Participant 5	23	171	68	Male

**Table 2:** Average fitting performance of the candidate models.

Model	G	GS	GC	GSC
$R^{2}$	0.667±0.095	0.731±0.068	0.675±0.098	0.739±0.069
RMSE	0.144±0.014	0.130±0.011	0.142±0.015	0.128±0.011
MAE	0.117±0.011	0.104±0.012	0.115±0.012	0.104±0.012

**Table 3:** Regression coefficients of the surface parameters.

	1	*v*	ϕ	$v^{2}$	vϕ	$\phi^{2}$
$T_{p}^{*}$	26.87	−36.98	−1.44	21.78	1.63	0.05
$\tau_{p}^{*}$	−0.51	1.35	0.05	−0.52	−0.04	0.00
$\sigma_{Tp}$	−2.38	4.56	0.28	−1.77	−0.22	−0.01
$\sigma_{\tau p}$	1.19	−1.07	−0.21	0.37	0.01	0.03
$\beta_{Tp}$	−11.43	25.05	0.73	−12.57	−0.81	−0.01
$\beta_{\tau p}$	−12.69	21.80	0.22	−9.34	−0.55	0.04

**Table 4:** Regression performance and LOOCV prediction errors of the surface parameters.

	$T_{p}^{*}$	$\tau_{p}^{*}$	$\sigma_{\mathrm{Tp}}$	$\sigma_{\tau p}$	$\beta_{\mathrm{Tp}}$	$\beta_{\tau p}$
Regression $R^{2}$	0.963	0.871	0.435	0.863	0.974	0.880
LOOCV MAE	0.813	0.022	0.102	0.339	0.184	0.563
LOOCV RMSE	1.067	0.028	0.122	0.421	0.252	0.755
Normalized MAE	12.98%	17.01%	33.45%	21.85%	9.33%	28.13%

**Table 5:** Candidate assistance patterns under different validation gait conditions.

Pattern	Parameter	$g_{v1}$	$g_{v2}$	$g_{v3}$	$g_{v4}$	$g_{v5}$
EO2	$T_{p}$ (%)	14.4	13.8	15.2	13.9	16.3
	$\tau_{p}$ (Nm/kg)	0.354	0.325	0.350	0.352	0.371
EO4	$T_{p}$ (%)	14.2	15.3	14.1	14.7	14.9
	$\tau_{p}$ (Nm/kg)	0.335	0.343	0.342	0.359	0.364
*M*	$T_{p}$ (%)	15.6	17.2	14.8	13.2	14.6
	$\tau_{p}$ (Nm/kg)	0.362	0.356	0.363	0.361	0.394
MO2	$T_{p}$ (%)	15.2	15.6	13.6	13.6	13.9
	$\tau_{p}$ (Nm/kg)	0.355	0.358	0.363	0.374	0.406

**Table 6:** Pairwise paired comparisons among the four assistance strategies.

Comparison	Mean Paired Difference	95% CI	*p*-Value
EO4–EO2	+7.7%	[−0.6,15.9]%	0.0614
*M*–EO2	+1.9%	[−5.8,9.5]%	0.5368
MO2–EO2	+6.4%	[−2.7,15.5]%	0.1233
*M*–EO4	−5.8%	[−14.6,3.0]%	0.1416
MO2–EO4	−1.3%	[−6.1,3.5]%	0.5071
MO2–*M*	+4.5%	[−0.9,10.0]%	0.0814

## Data Availability

The raw data supporting the conclusions of this article will be made available by the authors upon request.

## References

[1] Zhang J., Fiers P., Witte K.A., Jackson R.W., Poggensee K.L., Atkeson C.G., Collins S.H. (2017). Human-in-the-loop optimization of exoskeleton assistance during walking. Science.

[2] Slade P., Kochenderfer M.J., Delp S.L., Collins S.H. (2022). Personalizing exoskeleton assistance while walking in the real world. Nature.

[3] Kim J., Lee G., Heimgartner R., Arumukhom Revi D., Karavas N., Nathanson D., Galiana I., Eckert-Erdheim A., Murphy P., Perry D. (2019). Reducing the metabolic rate of walking and running with a versatile, portable exosuit. Science.

[4] Divekar N.V., Thomas G.C., Yerva A.R., Frame H.B., Gregg R.D. (2024). A versatile knee exoskeleton mitigates quadriceps fatigue in lifting, lowering, and carrying tasks. Sci. Robot..

[5] Lerner Z.F., Damiano D.L., Bulea T.C. (2017). A lower-extremity exoskeleton improves knee extension in children with crouch gait from cerebral palsy. Sci. Transl. Med..

[6] Herrin K.R., Pan Y.-T., Kesar T.M., Sawicki G.S., Young A.J. (2025). Robotic ankle exoskeleton and limb angle biofeedback for assisting stroke gait: A feasibility study. IEEE Robot. Autom. Lett..

[7] Afzal T., Zhu F., Tseng S.-C., Lincoln J.A., Francisco G.E., Su H., Chang S.-H. (2022). Evaluation of muscle synergy during exoskeleton-assisted walking in persons with multiple sclerosis. IEEE Trans. Biomed. Eng..

[8] Meagher M.A., Hunt G.R., Ortolano B., Murray R., Gabert L., Foreman K.B., Lenzi T. (2025). A biomechanical analysis of weighted lifting with an active knee exoskeleton. IEEE Access.

[9] Zhang Y., Jiang J., Ajoudani A., Tsagarakis N.G. (2026). Muscle fatigue-aware controller for a semi-rigid knee exoskeleton. IEEE Trans. Autom. Sci. Eng..

[10] Liu Z., Han J., Han J., Zhang J. (2024). Design and evaluation of a lightweight, ligaments-inspired knee exoskeleton for walking assistance. IEEE Robot. Autom. Lett..

[11] Zhang L., Liu G., Han B., Wang Z., Li H., Jiao Y. (2020). Assistive devices of human knee joint: A review. Robot. Auton. Syst..

[12] Nasiri R., Rayati M., Nili Ahmadabadi M. (2019). Feedback from mono-articular muscles is sufficient for exoskeleton torque adaptation. IEEE Trans. Neural Syst. Rehabil. Eng..

[13] Grimmer M., Zeiss J., Weigand F., Zhao G. (2022). Exploring surface electromyography (EMG) as a feedback variable for the human-in-the-loop optimization of lower limb wearable robotics. Front. Neurorobot..

[14] Jackson R.W., Collins S.H. (2015). An experimental comparison of the relative benefits of work and torque assistance in ankle exoskeletons. J. Appl. Physiol..

[15] Han H., Wang W., Zhang F., Li X., Chen J., Han J., Zhang J. (2021). Selection of muscle-activity-based cost function in human-in-the-loop optimization of multi-gait ankle exoskeleton assistance. IEEE Trans. Neural Syst. Rehabil. Eng..

[16] Wang W., Chen J., Ji Y., Jin W., Liu J., Zhang J. (2020). Evaluation of lower leg muscle activities during human walking assisted by an ankle exoskeleton. IEEE Trans. Ind. Inform..

[17] Ingraham K.A., Remy C.D., Rouse E.J. (2022). The role of user preference in the customized control of robotic exoskeletons. Sci. Robot..

[18] Beckerle P., Salvietti G., Unal R., Prattichizzo D., Rossi S., Castellini C., Hirche S., Endo S., Amor H.B., Ciocarlie M. (2017). A human–robot interaction perspective on assistive and rehabilitation robotics. Front. Neurorobot..

[19] Bryan G.M., Franks P.W., Song S., Voloshina A.S., Reyes R., O’Donovan M.P., Gregorczyk K.N., Collins S.H. (2021). Optimized hip–knee–ankle exoskeleton assistance at a range of walking speeds. J. Neuroeng. Rehabil..

[20] Franks P.W., Bryan G.M., Reyes R., O’Donovan M.P., Gregorczyk K.N., Collins S.H. (2022). The effects of incline level on optimized lower-limb exoskeleton assistance: A case series. IEEE Trans. Neural Syst. Rehabil. Eng..

[21] Shafer B.A., Powell J.C., Young A.J., Sawicki G.S. (2023). Emulator-based optimization of a semi-active hip exoskeleton concept: Sweeping impedance across walking speeds. IEEE Trans. Biomed. Eng..

[22] Durandau G., Rampeltshammer W.F., van der Kooij H., Sartori M. (2022). Neuromechanical model-based adaptive control of bilateral ankle exoskeletons: Biological joint torque and electromyogram reduction across walking conditions. IEEE Trans. Robot..

[23] Molinaro D.D., Kang I., Young A.J. (2024). Estimating human joint moments unifies exoskeleton control, reducing user effort. Sci. Robot..

[24] Hybart R.L., Ferris D.P. (2023). Neuromechanical adaptation to walking with electromechanical ankle exoskeletons under proportional myoelectric control. IEEE Open J. Eng. Med. Biol..

[25] Ding Y., Kim M., Kuindersma S., Walsh C.J. (2018). Human-in-the-loop optimization of hip assistance with a soft exosuit during walking. Sci. Robot..

[26] Monteiro S., Figueiredo J., Fonseca P., Vilas-Boas J.P., Santos C.P. (2024). Human-in-the-loop optimization of knee exoskeleton assistance for minimizing user’s metabolic and muscular effort. Sensors.

[27] Diaz M.A., Voss M., Dillen A., Tassignon B., Flynn L., Geeroms J., Meeusen R., Verstraten T., Babič J., Beckerle P. (2022). Human-in-the-loop optimization of wearable robotic devices to improve human–robot interaction: A systematic review. IEEE Trans. Cybern..

[28] Poggensee K.L., Collins S.H. (2021). How adaptation, training, and customization contribute to benefits from exoskeleton assistance. Sci. Robot..

[29] Koginov G., Bergmann L., Xiloyannis M., Rohner N., Ngo C., Duarte J.E., Leonhardt S., Riener R. (2023). Human-in-the-loop personalization of a bi-articular wearable robot’s assistance for downhill walking. IEEE Trans. Med. Robot. Bionics.

[30] Jing Z., Han H., Han J., Zhang J. (2024). A relationship model between optimized exoskeleton assistance and gait conditions improves multi-gait human-in-the-loop optimization performance. IEEE Trans. Neural Syst. Rehabil. Eng..

[31] Sun L., Jing J., Li C., Lu R. (2023). Multi-terrains assistive force parameter optimization method for soft exoskeleton. IEEE Trans. Neural Syst. Rehabil. Eng..

[32] Hansen N., Kern S. (2004). Evaluating the CMA evolution strategy on multimodal test functions. Proceedings of the International Conference on Parallel Problem Solving from Nature.

[33] Han J., Yin W., Song Y., Jing Z., Han J., Zhang J. (2026). A novel cable-tightness-based control strategy with high-accuracy and seamless gait assistance for cable-driven exoskeletons. IEEE Robot. Autom. Lett..

[34] Hermens H.J., Freriks B., Merletti R., Stegeman D., Blok J., Rau G., Disselhorst-Klug C., Hagg G. (1999). European recommendations for surface electromyography. Roessingh Res. Dev..

[35] Hermens H.J., Freriks B., Disselhorst-Klug C., Rau G. (2000). Development of recommendations for SEMG sensors and sensor placement procedures. J. Electromyogr. Kinesiol..

[36] Witte K.A., Fiers P., Sheets-Singer A.L., Collins S.H. (2020). Improving the energy economy of human running with powered and unpowered ankle exoskeleton assistance. Sci. Robot..

[37] Wang Z., Wei B., Ding Z., Zhu J., Zhang L., Zeng X., Yi C. (2025). Human-in-the-loop optimization for knee exoskeleton flexion assistance. IEEE Robot. Autom. Lett..

[38] Park D., An J., Lee D., Kang I., Young A.J. (2025). Human-in-the-loop optimization of hip exoskeleton assistance during stair climbing. IEEE Trans. Biomed. Eng..

[39] Zang W., Wu J., Wu J., Zhang Z., Wang S., Zhang Q. (2026). OpenSim–Umberger-based metabolic power stratification during the sit-to-walk transition using interpretable ensemble learning. Bioengineering.

[40] Ghai S. (2023). Novel methods for managing and assessing gait and posture in pediatric population. Children.

[41] Zhang X., Liu Y.-X., Wang R., Gutierrez-Farewik E.M. (2025). Multi-objective human-in-the-loop optimization of exoskeleton assistance for dropfoot gait. IEEE Robot. Autom. Lett..

[42] Chen Y., Miao S., Chen G., Ye J., Fu C., Liang B., Song S., Li X. (2024). Learning to assist different wearers in multitasks: Efficient and individualized human-in-the-loop adaptation framework for lower-limb exoskeleton. IEEE Trans. Robot..

[43] Zhang J., Cheah C.C., Collins S.H. (2017). Torque control in legged locomotion. Bioinspired Legged Locomotion.

[44] Liu Z., Yin W., Han J., Zhang J. Design and evaluation of a self-aligning knee exoskeleton for knee extension assistance during walking. Proceedings of the 2022 37th Youth Academic Annual Conference of Chinese Association of Automation (YAC).

